# An Ultrasound Evaluation of the Vertebral Artery in Patients With Vertebral Artery Hypoplasia

**DOI:** 10.7759/cureus.15020

**Published:** 2021-05-14

**Authors:** Sina Zarrintan, Joe Iwanaga, Mehrdad Mozafar, Abolhassan Shakeri-Bavil, Mohamad Mozafar, R. Shane Tubbs

**Affiliations:** 1 Department of General & Vascular Surgery, Shahid Beheshti University of Medical Sciences, Tehran, IRN; 2 Department of Neurosurgery, Tulane University School of Medicine, New Orleans, USA; 3 Faculty of Medicine, Tehran University of Medical Sciences, Tehran, IRN; 4 Department of Radiology, Tabriz University of Medical Sciences, Tehran, IRN

**Keywords:** vertebral artery, hypoplasia, ultrasound, vertebrobasilar insufficiency

## Abstract

Purpose

The aim of the current study was to assess and compare Doppler ultrasound findings, especially the resistivity index (RI), among and between patients with vertebral artery hypoplasia (VAH) and normal populations.

Material and methods

Fifteen consecutive patients with VAH (mean age 54 ± 21 years) and 15 sex-matched controls without VAH (mean age 54 ± 22 years) were selected for the study. The vertebral arteries (VA) were examined with Doppler ultrasound. We also explored each group for sex and age differences (young: age ≤ 50, old: age >50).

Results

The mean RI (MRI), right RI (RRI), left RI (LRI), non-dominant-side RI, and dominant-side RI were significantly higher in the Case Group than the Control Group. In the Case Group, the affected-side RI (A.RI) was significantly higher than the normal side, while the normal side peak systolic velocity was significantly higher than the affected side. The MRI and A.RI were significantly higher in older patients. We also found a significant negative correlation between the mean diameter (MD) and MRI. MRI and A.RI both correlated positively with age in the Case Group, while left peak systolic velocity decreased significantly with age in the Control Group [p-values < 0.05].

Conclusion

The dominant VA had a higher RI in the Case Group than the Control Group. It can therefore be inferred that the dominant VA in patients with VAH does not work completely normally, thus making these patients even more susceptible to vertebrobasilar insufficiency and possible strokes.

## Introduction

The vertebral artery (VA) is the main artery supplying infratentorial structures such as the cerebellum and medulla; its diameter is determined genetically [1]. Variant luminal diameters of the VA range from asymmetry to an even more severe difference, a hypoplastic VA. There seems to be VA asymmetry, defined as a side-to-side diameter difference of 0.3mm, in some 68.9% of the population, the left side VA being dominant [2]. Vertebral artery hypoplasia (VAH) is a relatively common congenital variation. The VA diameter is small and there are increases in the ipsilateral resistivity index (RI) (RI ≥ 0.75) and the contralateral diameter (side-to-side diameter difference ≥ 1.2mm), and a decrease in flow volume [2].

The lack of general consensus concerning the exact cut-off value for a hypoplastic VA diameter, along with the use of different modalities to assess the VA, has led to a wide spectrum of reported VAH prevalences in the literature. The reported frequencies of unilateral VAH range from 2.1% to 26.5%, right side VAH being more frequent [3,4]. Proposed cut-off values for a hypoplastic VA have ranged from 2mm [5] to 3mm [6] in previous studies.

The clinical importance of VAH is not well recognized. Two previous studies found no signs of vertebrobasilar insufficiency (VBI) in patients with VAH [5,7]. They, therefore, proposed that VAH could be a normal variant in the normal population because of appropriate contralateral side compensation. Nevertheless, VAH has received increasing attention in recent years and studies have revealed that it is not just an innocent vascular variant. It can present with signs and symptoms of VBI, especially when the dominant VA fails to supply sufficient blood to the posterior circulation or when it is accompanied by other vascular risk factors [8]. Different studies have concluded that VAH is significantly more prevalent in patients with posterior circulation stroke than in those with anterior circulation stroke, suggesting that it is a predisposing factor for the former [1,2,9-12]. This makes investigations into VAH much more important.

The VA has received much less attention than the carotid artery because its anatomical location makes it difficult to evaluate by Doppler ultrasound, which is one of the easiest and most routine tools for assessing vessels. Its main advantage is that it is non-invasive and harmless and can be repeated several times. Some studies have measured the Doppler parameters in VAH in addition to their main goals. An important limitation of those studies is that few of them have measured Doppler parameters in normal individuals without VAH. Also, to our knowledge, no study has compared RI between patients with VAH and normal subjects without VAH.

The aim of the current study was to assess and compare statistical differences in power Doppler parameters between patients with VAH and normal populations, along with side-to-side differences in each group.

## Materials and methods

Ethical considerations

We conducted the present study in the Section of Angiography, Department of Radiology and Radiotherapy, Imam Reza Hospital, Tabriz University of Medical Sciences, Tabriz, Iran, from January 2013 to December 2014. Informed consent was obtained from all participants.

Patients and variables

All patients who had a documented VAH in Doppler ultrasound studies were considered for the study. We selected those over 18 years of age. The exclusion criterion was any history of documented cerebrovascular events resulting from anterior circulation pathologies. Fifteen consecutive patients with VAH were selected according to this criterion; they had a hypoplastic VA on either the left or the right side. We also selected 15 normal individuals as the Control Group and performed Doppler ultrasound studies on the VAs of both sides. The VAs were imaged in the mid-cervical segment (V2) in the supine position after at least four pulse wave cycles. A single operator performed all the examinations. We defined VAH as a VA diameter of 2.1mm or less in Doppler ultrasound.

We recorded the age and sex of each subject in the case and Control Groups. We also divided the subjects into two groups on the basis of age: (1) Age ≤ 50, defined as "young" (n=5 in Case Group, n=5 in Control Group), (2) Age>50, defined as "old" (n=10 in Case Group, n=10 in Control Group). The Doppler ultrasound data were Peak Systolic Velocity (PSV), RI, and VA diameter. We calculated the RI from the following formula: (peak systolic velocity - end-diastolic velocity)/peak systolic velocity. These parameters were measured on both sides. We calculated the mean PSV, RI, and VA diameter for the Case and Control Groups.

Statistical analyses

We had four sets of Doppler ultrasound parameters containing measured and calculated ultrasound parameters of the VA on each side for both the Case and Control Groups. SPSS software version 22 (IBM inc., Armonk, NY, USA) was used for all statistical analyses. We used independent-sample t-test, paired-sample t-test, Wilcoxon test, and Man-Whitney U test to determine the significance of any difference in the Doppler ultrasound parameters between the Case and Control Groups, and the side-to-side differences in each group; and also to identify statistical differences in any of those parameters in relation to sex or age. The correlations of all parameters with age and vessel diameter were determined using Spearman’s rank correlation coefficient. We considered p-values less than 0.05 to be statistically significant.

## Results

We selected 15 patients with a documented history of VAH on either the left or the right side for the Case Group, and 15 healthy individuals with normal VAs for the Control Group. Thus, 60 VAs were examined and their Doppler parameters were compared.

Within-group and between-group analyses

Table 1 gives the descriptive statistics and frequency of background variables for the study patients. These background variables did not differ significantly between the Case and Control Groups. In the whole study population, there were no correlations between parameters except for a significant negative correlation between mean diameter (MD) and mean RI (MRI) (R=-0.613).

**Table 1:** Background characteristics of Case and Control Groups. *Dichotomous variables and scale variables are presented as number (%) and mean±standard deviation respectively. MD - Mean Diameter; MPSV - Mean Peak Systolic Velocity; MRI - Mean Resistivity Index; RRI - Right Resistivity Index; LRI – Left Resistivity Index; RPSV – Right Peak Systolic Velocity; LPSV – Left Peak Systolic Velocity; RD – Right Diameter; LD – Left Diameter.

Variable	Case*	Control*	Total*	p-value
Sex				1
Male	13 (86.5%)	12 (80%)	25 (83.3%)	
Female	2 (13.3%)	3 (20%)	5 (16.7%)	
Categorized age				1
Young (<50 years)	5 (13.3%)	5 (13.3%)	10 (33.3%)	
Old (>50 years)	10 (66.7%)	10 (66.7%)	20 (66.6%)	
Age	54±21 years	54±22 years	54±21	0.94
MRI	0.79±0.04	0.68±0.06	0.74±0.07	p<0.05
MPSV	35.80±8.31	41.60±15.06	38.70±12.31	p=0.561
MD	2.80±0.24	3.45±0.33	3.12±0.43	p<0.05
RRI	0.81±0.06	0.70±0.06	0.75±0.08	p<0.05
LRI	0.77±0.05	0.67±0.07	0.72±0.08	p<0.05
RPSV	35±9	40±14	37±12	p=0.36
LPSV	37±11	43±21	40±17	p=0.57
RD	2.5±0.8	3.2±0.4	2.8±0.7	p<0.05
LD	3.1±0.9	3.7±0.7	3.4±0.9	p=0.088
Non-dominant RI	0.82±0.06	0.69±0.07	0.76±0.09	p<0.05
Dominant RI	0.75±0.04	0.68±0.06	0.72±0.07	p<0.05
Non-dominant PSV	32±9	39±14	36±12	p=0.163
Dominant PSV	39±10	44±20	42±16	p=0.835
Non-dominant Diameter	2±0.1	3±0.3	2.5±0.6	p<0.05
Dominant Diameter	3.6±0.5	3.9±0.6	3.8±0.6	p=0.228

**Figure 1:**
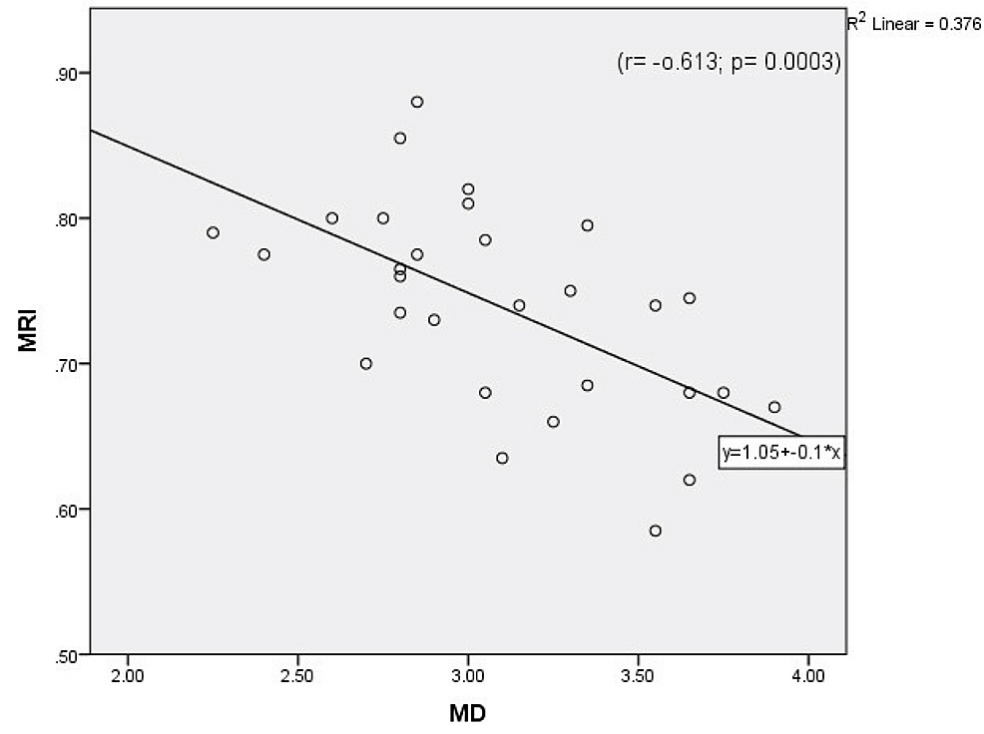
Correlation between mean diameter (MD) and mean resistivity index (MRI) in the whole study population.

**Figure 2:**
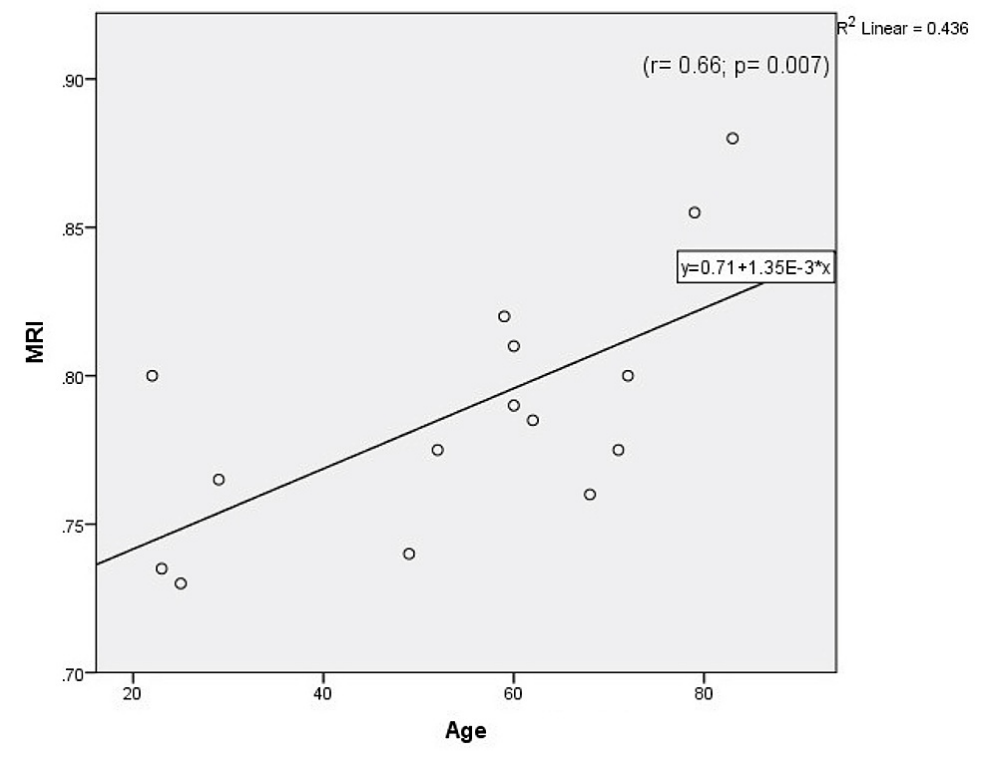
Correlation between age and mean resistivity index (MRI) in the Case Group.

**Figure 3:**
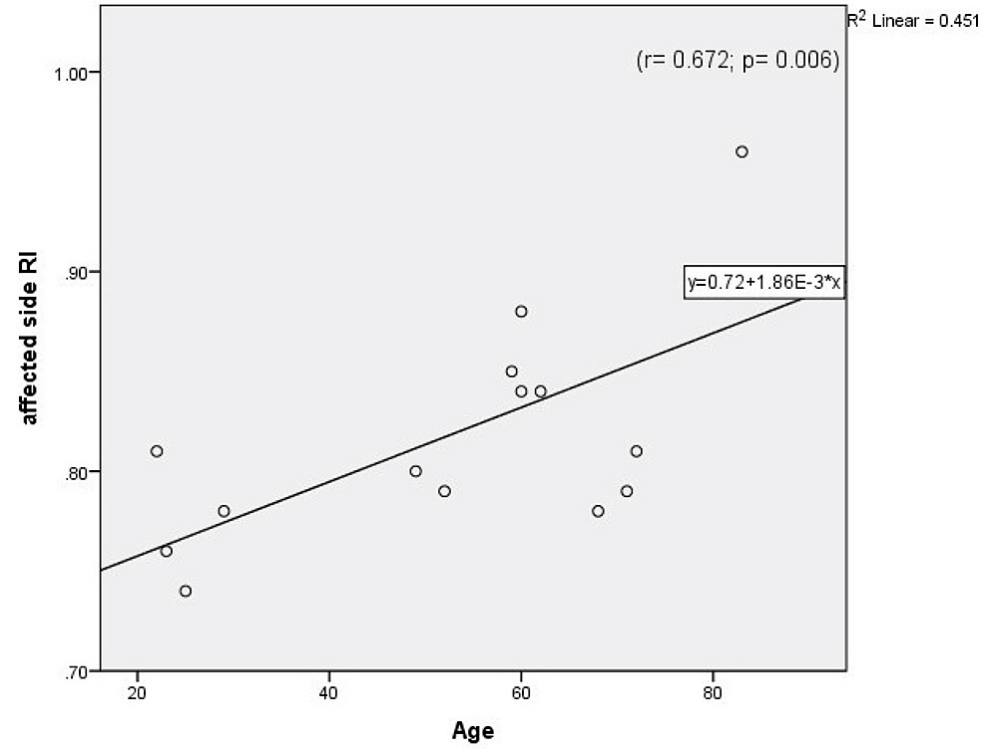
Correlation between age and affected side (hypoplastic side) resistivity index in the Case Group.

**Figure 4:**
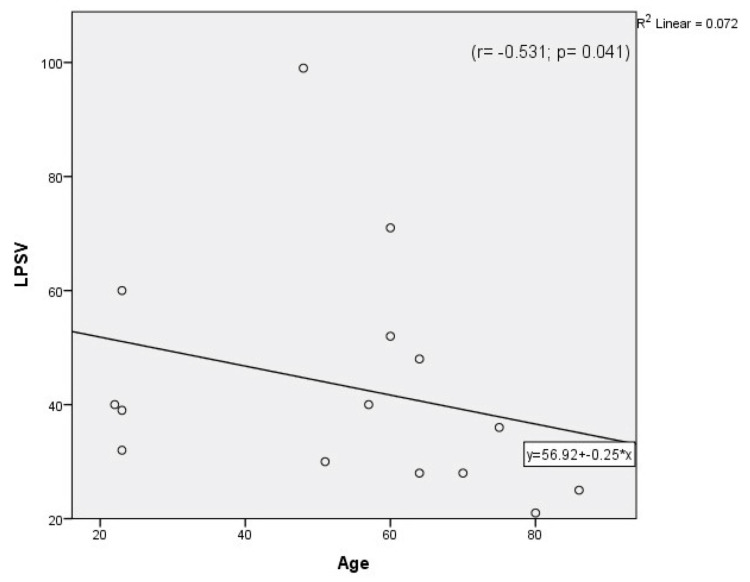
Correlation between age and left peak systolic velocity (LPSV) in the Control Group.

Table 1 compares both calculated and measured parameters between the Case and Control Groups. The MRI was significantly higher in the Case Group than the Control Group, and the MD of the Control Group was significantly higher than that of the Case Group. However, the difference between the two groups in mean peak systolic velocity (MPSV) was not significant [p-values=0.000003, 0.000001, 0.561 respectively]. As can be seen in Table 1, the right resistivity index (RRI) and left resistivity index (LRI) were significantly higher in the Case Group than the Control Group [p-alue< 0.05]. Also, the right-side diameter (RD) and non-dominant-side diameter were significantly greater in the Control Group [p-value<0.05]. Moreover, dominant- and non-dominant-side RIs were significantly higher in the Case Group than the Control Group. There were no correlations between age and the measured and calculated parameters in either group except that both MRI and the affected side RI (A.RI) correlated positively with age in the Case Group, while the left-side PSV (LPSV) decreased significantly with age in the Control Group (Figures 1-4)

As expected, the A.RI was significantly higher than the normal-side RI in the Case Group, and the PSV and diameter were significantly higher on the normal side than the affected side [p-values=0.000087, 0.013, and 0.001, respectively] (Table 2).

**Table 2:** Side-to-side differences between left and right and dominant and non-dominant parameters in each group. The presented values show either side-to-side difference or dominant and non-dominant difference of the values presented in Table 1, i.e., the mean difference of each value between left and right side or dominant and non-dominant side among Case and Control Groups. Values are presented by mean±standard deviation. Negative values show that the parameters are lower on the left side than on the right side or lower on the dominant side than the non-dominant side. *Indicates that there is a significant difference (p-value<0.05) between right and left sides or dominant and non-dominant sides for that value. PSV – Peak Systolic Velocity; RI – Resistivity Index.

Group	Left side vs. right side			Dominant side vs. non-dominant side		
	diameter	PSV	RI	diameter	PSV	RI
Case	0.7± 1.7	2±12	-0.03±0.08	1.7±0.5*	7±10*	-0.07±0.05*
Control	0.5±1	3±18	-0.03±0.06	0.9±0.6*	4±18	-0.02±0.07

​Subgroup analyses

In the Case Group, there were no significant differences in the affected or normal side parameters between males and females or between young and old subjects, except that the A.RI was significantly higher in the old than the young group [0.8430 vs. 0.7780, p-value=0.031] (Table 3).

**Table 3:** Comparison between male and female patients in presented data. All data are presented as mean±SD. *Indicates significant differences between males and females in each group (p-value<0.05). MD – Mean Diameter; MPSV – Mean Peak Systolic Velocity; MRI – Mean Resistivity Index; RI – Resistivity Index; PSV: – Peak Systolic Velocity.

Variable	Case group		Control group	
	Female	Male	Female	Male
MRI	0.79±0.02	0.79±0.05	0.68±0.06	0.68±0.06
MPSV	35.75±5.30	35.81±8.85	58.33±14.87*	37.42±12.37
MD	2.58±0.25	2.83±0.22	3.48±0.36	3.44±0.34
Non dominant RI	0.80±0.01	0.82±0.06	0.66±0.07	0.70±0.07
Dominant RI	0.78±0.02	0.75±0.04	0.70±0.06	0.67±0.04
Non dominant PSV	38.0±9.9	31.2±9.0	58±11*	35±11
Dominant PSV	33.50±0.71	40.38±10.78	59±18*	40±20
Non dominant diameter	2.00±0.00	1.95±0.07	2.9±0.2	3±0.3
Dominant diameter	3.2±0.5	3.7±0.5	4±0.8	3.8±0.6

Also, the MRI was significantly higher in the old patients (age>50) [0.8050 vs.0.7540, p-value=0.021] (Table 4).

**Table 4:** Comparison between young and old patients in presented data. All data are presented as mean±standard deviation. *Indicates significant differences between the young and the old in each group (p-value<0.05). MD – Mean Diameter; MPSV – Mean Peak Systolic Velocity; MRI – Mean Resistivity Index; PSV – Peak Systolic Velocity; RI – Resistivity Index.

Variable	Case group		Control group	
	Old (age >50)	Young (age ≤50)	Old (age >50)	Young (age ≤50)
MRI	0.80±0.04*	0.75±0.03	0.68±0.05	0.69±0.06
MPSV	33.95±8.38	39.50±7.62	39.25±15.31	46.30±14.98
MD	2.78±0.26	2.85±0.20	3.44±0.36	3.46±0.32
RI of affected side	0.84±0.06*	0.78±0.03	0.70±0.06	0.68±0.08
RI of non-affected side	0.77±0.03	0.73±0.04	0.66±0.07	0.70±0.05
PSV of affected side	31.1±10.4	34.2±6.1	39±16	41±12
PSV of non-affected side	36.80±9.99	44.80±9.58	40±16	52±27
Diameter of affected side	1.97±0.05	1.94±0.09	3±0.3	3.1±0.2
Diameter of non-affected side	3.6±0.5	3.8±0.5	3.9±0.6	3.8±0.7

The only significant difference between males and females in the Control Group was that MPSV, right-side PSV (RPSV), non-dominant side PSV, and dominant side PSV were remarkably higher in women (Table 3). There were no significant differences between young and old individuals in any of the measured or calculated parameters in this group (Table 4).

Of the 15 patients in the Case Group, 10 had VAH on the right side and five on the left. The 15 individuals in the Control Group had no VAH on either side. Table 5 compares the right- and left-side parameters in each group. Among the patients with right-side VAH, RRI and left-side diameter (LD) were significantly greater than the contralateral side parameters [p-values=0.001 and 0.005, respectively]. Among those with left-side VAH, LRI and RD were significantly higher than their contralateral counterparts [p-values=0.03 and 0.043, respectively]. There were no significant differences between the right- and left-side parameters in the Control Group (Table 5).

**Table 5:** Comparison of ultrasound parameters between right- and left-side VA in each group. *Presented as mean±standard deviation. VAH – Vertebral Artery Hypoplasia; VA – Vertebral Artery; PSV – Peak Systolic Velocity; RI – Resistivity Index.

Group	Variable	Right-side parameter*	Left-side parameter*	Total*	p-value
Patients with right side VAH	RI	0.83±0.07	0.75±0.04	079±0.05	p<0.05
	PSV	35.2±9.4	42.4±9.28	38.80±7.57	p=0.068
	Diameter	1.98±0.07	3.7±0.4	2.58±0.2	p<0.05
Patients with left side VAH	RI	0.77±0.02	0.81±0.03	079±0.02	p<0.05
	PSV	35±11	26±4	29.8±6.73	p=0.078
	Diameter	3.4±0.6	2±0.1	2.70±0.29	p<0.05
Control group	RI	0.70±0.06	0.67±0.07	0.68±0.06	p=0.117
	PSV	40±14	43±21	41.6±15.06	p=0.727
	Diameter	3.2±0.4	3.7±0.7	3.45±0.33	p=0.082

We also compared the measured and calculated ultrasound parameters between patients with right-side VAH and those with left-side VAH. Table 6 shows no significant differences in these parameters between those two patient subgroups, except that MPSV was significantly higher in patients with right-side VAH [p-value=0.043] (Table 6).

**Table 6:** Comparison of ultrasound parameters between patients with right-side VAH and patients with left-side VAH. *Presented as mean±standard deviation MRI – Mean Resistivity Index; MPSV – Mean Peak Systolic Velocity; MD – Mean Diameter; RI – Resistivity Index; PSV – Peak Systolic Velocity; VAH – Vertebral Artery Hypoplasia

Variable	Patients with right-side VAH*	Patients with left-side VAH*	Total*	p-value
MRI	0.79±0.05	0.79±0.02	0.79±0.04	p=0.902
MPSV	38.80±7.57	29.80±6.73	35.80±8.31	p=0.043
MD	2.85±0.20	2.70±0.29	2.80±0.24	p=0.262
Affected side RI	0.83±0.07	0.81±0.03	0.82±0.06	p=0.738
Non-affected side RI	0.75±0.04	0.77±0.02	0.75±0.04	p=0.434
Affected side PSV	35.2±9.4	26±4.3	32.1±9.1	p=0.061
Non-affected side PSV	42.4±9.28	33.6±10.55	39.47±10.27	p=0.121
Affected side Diameter	1.96±0.7	1.96±0.05	1.96±0.06	p=0.825
Non-affected side Diameter	3.7±0.4	3.4±0.6	3.6±0.5	p=0.295

.

## Discussion

We found hypoplastic VA more frequently on the right side (66.7%), in agreement with previous studies [3,4,9,12-14]. The right VA was the non-dominant VA in most of our control patients, also in agreement with previous studies [13,15]. The left VA has proved dominant in most whole study populations [2,5,6,16-20], though Scheel et al. [7] found no significant side-to-side differences in VA diameters among 78 healthy adults. This left-side dominance could be related to the branching of the left VA from the left subclavian artery, which derives directly from the aortic arch, so it undergoes higher shear stress. Theirfelder et al. [13] found that both RD and LD were significantly smaller in the VAH group than the non-VAH group, but only RD was significantly smaller in the VAH group in our study. The main difference between the Theirfelder et al. [13] study and ours is that their non-VAH subjects were admitted because of a suspected stroke while the non-VAH group in our study was selected from the normal population.

The VA diameters in our Control Group were consistent with previous studies [15,17,18], though there is one report of much greater VA diameters, 4.43±0.75 mm on the right side and 4.58±0.76 mm on the left [19]. However, in neither study were patients with VAH distinguished from the other patients for separate analyses. Also, none of these studies distinguished normal VAs, except for Kizilkilic et al. [15], who verified their VAs as normal by angiography; however, their patients had concurrent carotid artery stenosis. The latter could have affected the VA diameters owing to a compensatory mechanism since the RD was significantly larger in patients with internal carotid stenosis >50% than in those with internal carotid stenosis <50%; though other parameters (LD, RPSV, LPSV) did not differ significantly between those two groups. 

Previous studies showed that the VA diameter on each side and also the mean VA diameter were significantly lower in women than men [2,16-18,20,21], though Kizilkilic et al. [15] found no sex difference in right side diameters, and, as in our study, LD was not significantly lower in women. Surprisingly, although there were far fewer women than men in the present study’s Control Group, the mean VA diameter and RD tended to be larger in women. Scheel et al. [7] found that, in contrast to previous studies, males and females had similar mean VA diameters [15,17,19].

Some studies have revealed an increase in VA diameter with increasing age [7, 21]. Chen et al. [20] found a mild positive correlation between age and left VA diameter. In contrast, Jeng et al. [2] found a positive correlation between age and right side VA. However, Schoning et al. [5], in a study of 94 healthy children and adolescents, found that in contrast to other major cervical vessels the VA diameter remained constant with increasing age. There was no correlation between age and VA diameter on either side among all subjects in that study, or in any group of subjects in the present study.

The MPSV in our Control Group is consistent with previous studies, although some investigators have demonstrated a much higher PSV in their normal subjects [15-17,22]. However, there were patients with VAH among the healthy subjects in previous studies, except that by Kizilkilic et al. [15], in which the patients' VAs were confirmed normal by angiography. We found that the LPSV was higher than the RPSV in subjects with normal VAs but the difference was not significant, which is consistent with Kizilkilic et al. [15]. However, some investigations have shown significant left dominance for PSV [2,18,20]. The mean RI in our Control Group is consistent with previous studies, although again there were individuals with VAH among their healthy subjects [17,22]. There were no significant differences in RI or diameter between male and female subjects in our Control Group, in agreement with a previous study; though in contrast to that study, we found that MPSV, RPSV, dominant PSV, and non-dominant PSV were significantly higher in women, as others have reported [2,15, 20] This was unexpected because there were far fewer women than men in the present study. Jeng et al. [2] evaluated 447 subjects who had non-specific neurological complaints or were receiving physical check-ups and found that women had significantly lower RI than men. Moreover, in the Chen et al. study [20], the left but not the right RI was significantly lower in women than men in a population of 1000 healthy individuals receiving physical check-ups including cervical ultrasound examinations. 

Some studies have revealed an age-dependent decline in PSV in healthy subjects [5,7]. Likewise, some studies showed negative correlations between each side's PSV and age [2,20]. However, we found a negative correlation between PSV and age only for the left side VA in the Control Group. Nevertheless, the main difference between previous studies and the present one is that our healthy subjects (Control Group) had totally normal VAs, while in previous studies there were subjects with VAH among the healthy individuals. We also found that both MRI and A.RI correlated positively with age in our Case Group. One study reported a significant increase in MRI with age in their 78 healthy adults, although again those 78 included some subjects with VAH [7]. However, Chen et al. found no significant correlation between age and RI in their 1000 subjects, with a VAH frequency of 9.5%.20 Besides, unlike our study, they noted a significant negative correlation between each side diameter and RI [20]. There were no correlations between MD, MRI, and MPSV in the total study population or the Case or Control Group, except that MD correlated negatively with MRI in the total study population, which is consistent with previous publications [2] 

We grouped the subjects with VAH (case group) into young and old subgroups, but there were no significant differences between them except that MRI and A.RI were significantly higher in the older patients. Older patients with VAH should receive more attention, not only because of their high-resistance VAs but also because RI increases with age, as our study showed. However, in the Control Group (normal VAs), the young and old patients did not differ significantly in any of the measured and calculated parameters, which is consistent with the Kizilkilic et al. study [15] Likewise, Seidel et al. [18] divided their study population into young (<55 years) and old (>55 years), though their cut-off point for age was a little different. They found no significant differences in VA diameter or PSV between their young and old groups. Schoning et al. [5] noted that healthy adults (age over 20 years) had significantly lower PSV and RI than healthy adolescents (age range 10-18 years).

Vertebrobasilar insufficiency related to the insufficient blood supply to the posterior circulation of the brain due to stenosis and occlusion of the VA can cause symptoms such as dizziness, vertigo, numbness, double vision, and drop attacks [23,24]. In view of the nonspecific and subjective clinical manifestations of VBI, Doppler examination is of great importance for screening patients and for detecting the underlying defect. Although arteriography is the gold standard tool for detecting VA disease, it is only used for patients with indications and is not appropriate for screening owing to its invasiveness and high cost. Complications include arterial puncture, allergic and hypotensive reactions, and subintimal injection or elevation of atherosclerotic plaques by the catheter tip [25]. Doppler ultrasound is cost-effective, non-invasive, and widely accessible in more clinical centers. It is much more dependent on sonographer skills, but it is nevertheless the most frequently used method for imaging the posterior circulation vessels. Some previous studies have shown a correlation between Doppler sonography and VA arteriography [26-28]. Nicolau et al. [28] found a sensitivity of 90%, a specificity of 100%, a positive predictive value of 100%, and a negative predictive value of 95% for detecting vertebrobasilar disease with Doppler ultrasound when they examined 116 VAs. Magnetic resonance angiography (MRA) can also be useful for evaluating the vertebrobasilar system, although small and peripheral branches of VAs can be poorly visualized by this procedure [29]

Previous studies have shown that VAH is more frequent in posterior circulation infarction (PCI) than anterior circulation infarction (ACI), suggesting a topographical preponderance of brainstem-cerebellar infarctions in patients with VAH [3,4], implying that such patients are at increased risk for strokes in this region. Our study showed no difference between the ages of the case (patients with VAH) and control (subjects without VAH) groups. However, previous studies assessed patients with ischemic stroke and found that those with VAH were significantly younger, suggesting that VAH advances the timing of first brain strokes [12,14]. Another study showed VAH to be common even in young subjects with PCI, suggesting it as an independent risk factor for PCI in those patients [11]. This makes the diagnosis of VAH more important even in young individuals, although VAH is less of a risk for PCI than other independent risk factors in young subjects [11]. The exact pathomechanism of these ischemic strokes is still under investigation. 

Seidel et al. [18] evaluated 50 hospitalized patients with nonvascular diagnoses. They noted that a smaller right-side VA diameter led to lower flow volume on that side; however, they did not identify VAH patients among their subjects. Another study showed relative regional hypoperfusion of the PICA territory in CT perfusion maps among 42.4% of their VAH patients, significantly more than in their non-VAH patients [13]. Moreover, a previous study demonstrated a significantly reduced flow volume in hypoplastic VAs, which can lead to regional hypoperfusion especially when the dominant VA fails to compensate [20]. Therefore, although the contralateral side VA can have a significantly higher compensatory flow volume [5,20], it will not necessarily compensate completely for the hypoplastic side. Thus, the net VA flow volume could be lower in patients with VAH than in subjects without VAH [3,20]. Likewise, we found that the dominant VA had a surprisingly higher RI in the Case Group than the Control Group. Therefore, it could be inferred that the dominant VA in patients with VAH is not completely normal either, making such patients even more susceptible to VBI and subsequent strokes. In agreement with this inference, a previous study demonstrated that dominant VA stenosis was more frequent in patients with VAH than those without, consistent with the assumption that their dominant VA was abnormal [12]. Other possible stroke mechanisms have also been suggested in these patients, including atherosclerotic and thrombotic processes in hypoplastic VA, and an embolic mechanism due to the dominant VA having a compensatory diameter large enough to accommodate the embolus [4,9].

The present study had some limitations. First, the sample size was small. Second, because there is no single accepted definition of VAH, other cut-offs for VA diameter could yield different results, though we considered one of the lowest cut-offs in order to achieve greater specificity. Our biggest advantage is that we selected the non-VAH group from a normal population and we compared their ultrasound data with those from VAH patients. Also, to our knowledge, this is the first study to focus on differences in Doppler parameters, especially RI, between a normal population without VAH and subjects with VAH [13, 30]. Previous studies have mostly been conducted on East Asian or Western populations; to the best of our knowledge, this is the first study on a West Asian population. Owing to racial-ethnic differences, caution is required in extrapolating our findings to other populations.

## Conclusions

The dominant VA has higher RI in patients with VAH than in normal subjects without VAH. Therefore, it could be inferred that the dominant VA in patients with VAH does not work completely normally, so these patients are even more susceptible to vertebrobasilar insufficiency and possible strokes. Future studies on larger populations and with CT angiography (CTA) or MRA are needed to analyze and compare VA waveforms, especially for abnormalities such as VAH.
